# Evaluation of highway debris flow hazard based on geomorphic evolution theory coupled with material response rate

**DOI:** 10.1038/s41598-024-64279-y

**Published:** 2024-06-12

**Authors:** Na He, Ruze Han, Guisheng Hu, Zhiquan Yang, Linjuan Xu, Filip Gurkalo

**Affiliations:** 1 School of Civil Engineering, Henan Polytechnic University, Jiaozuo, 454000 China; 2 GongQing Institute of Science and Technology, Gongqingchengshi, 332020 China; 3 State Key Laboratory of Earthquake Dynamics, Institute of Geology, China Earthquake Administration, Beijing, 100029 China; 4 Institute of Mountain Hazards and Environment, Chinese Academy of Sciences and Ministry of Water Resources, Chengdu, 610041 Sichuan China; 5 Faculty of Public Safety and Emergency Management, Kunming University of Science and Technology, Kunming, 650093 China; 6 Key Laboratory of Lower Yellow River Channel and Estuary Regulation, MWR, Yellow River Institute of Hydraulic Research, YRCC, Zhengzhou, 450003 China

**Keywords:** Highway debris flow, Gully information entropy, Physical source sensitivity, Watershed unit material response rate, Hazard evaluation, Environmental sciences, Natural hazards

## Abstract

Assessments of highway feasibility frequently lack the detailed data and geological information necessary to conduct hazard evaluations of debris flows. This study discusses the processes of debris flow development when regional rainfall meets the critical level required for debris flow initiation. It utilizes geomorphic evolution theory and establishes a regional risk assessment matrix for debris flow by combining information about gullies and source sensitivity. Considering the location relationship between the highway and debris flow gullies, a rapid evaluation method for debris flow risk assessment along the G318 highway in Sichuan Province is proposed by modifying the judgment matrix. The four debris flow gullies constructed during the upgrading project in Yajiang County, stretching from the west of the city to the Shearer Bay section, were analyzed via examples. The results show that, among the four selected debris flow gullies, two had medium hazard levels, and two had high hazard levels. The validation results are consistent with the actual results, implying that the evaluation method used in this study is accurate and feasible. This method is suitable for the rapid evaluation of debris flow disaster hazards in the feasibility assessment stage of a highway because it relies on readily available data sources, and the evaluation results are fast and convenient. The highway passes through four debris flow gullies, which directly impact the alignment of this particular section of the geological route and the engineering layout. Based on current specifications, the maximum impact range of a one-time debris flow under the given frequency conditions was calculated using the "rainfall method." The results showed that the maximum impact ranges of a debris flow, occurring once in 100 years, for four gullies would be 9.08 m, 9.09 m, 10.86 m, and 10.08 m. The safe clearance heights of bridges over the four gullies should be 14.58 m, 14.59 m, 16.36 m, and 16.3 m. Additionally, the safety clearance width for all gullies should be 5.0 m.

## Introduction

With its vast territory and complex geological conditions, China shows some of the most severe geological hazards, with the most significant number of people at risk. In recent years, geologic disasters induced by strong earthquakes and extreme meteorological events have paralyzed regional highway traffic, resulting in substantial losses of national property and people's lives and seriously affecting the ecological balance in the affected areas^1^. According to China’s Natural Resources Statistical Bulletin, 5659 geologic disasters occurred in 2022, with direct economic losses of CNY 1.50 billion^2^. Highway transportation occupies a strategic position in the national economy. Constructing high-speed infrastructure on remote routes is a typical economically orientated behavior with positive externalities. In contrast, highway debris flow disasters, as one of the main types of highway geologic hazards, have the characteristics of suddenness and destructiveness. Once a debris flow occurs, it will block traffic and damage vehicles in non-severe cases, but in heavy cases, it will result in injuries and deaths and threaten people's lives and properties. In October 2009, prolonged heavy rainfall in the northeastern region of Sicily, covering about 50 km^2^, triggered hundreds of landslides (mainly debris flow), sweeping over the highest points of many villages, crossing the SS114 state highway and the Messina–Catania railway, killing more than 30 people^3^. In July 2013, a cluster of debris flowing along the mainstream of the Minjiang River in Wenchuan County, Sichuan Province, caused significant damage to bridges, roadbeds, and tunnels on the Duwen Highway (Line G213) and the Duwen Expressway, disrupting the Line G213 and the Duwen Expressway at a total of 16 locations^4^. In April 2016, as a result of heavy rainfall, 16 people were killed by a cluster of debris flowing along the Karakorum Highway in Pakistan, and the Chuchang and Kiyal Bridges were destroyed entirely, with debris flow blocking traffic for several hours^5^. In August 2019, a large-scale debris flow disaster occurred in Dengxi gully in Mianwang Town, Wenchuan County, leading to the destruction of houses at the mouth of the gully, as well as of national highways and expressways, and the disruption of transportation for months, while a large amount of washed-out material blocked the Minjiang River channel, diverting it to the left bank and raising its water level, creating a chain of disasters^6^. In August 2020, a debris flow occurred in Gongbu Jiangda County, Linzhi City, Tibet Autonomous Region, burying the G318 road up to 53 m and causing traffic congestion^7^. These cases show that highway construction faces severe challenges, and it is necessary to strengthen disaster prevention and mitigation and improve the disaster-resistant capacity of highways to ensure their safety and smoothness.

The G318 National Highway runs from the People’s Square in Huangpu District, Shanghai, to the Friendship Bridge in Rikaze City, Nyalamu County, China–Nepal, stretching for 5476 km. A section from Yajiang County to the Jianziwan Tunnel is proposed as part of a quality improvement project for the G318 line in the transition zone from the Qinghai–Tibet Plateau to the Sichuan Basin. The area is steep and part of a highly mountainous canyon region. The region has unique climate conditions, rainfall characteristics, topographic geomorphological features, deep regional fractures, and strong crustal uplift. Debris flows have developed, resulting in severe disasters and serious risks in this highly active region. Therefore, the transportation routes through this region are subjected to severe threats, resulting in vast economic losses and casualties^8^. The proposed highway passes through the middle and lower reaches of the Gesse Gulch watershed, which is a debris flow circulation accumulation area. The wide distribution, type, and quantity of loose solids in the watershed and the relatively abundant rainfall conditions in the region make it more likely that debris flow will occur here. In such an event, the construction and operation of the proposed highway route will be jeopardized.

Most hazard evaluation methods addressing regional highway debris flow use geomorphic information entropy, artificial neural networks, and multi-factor synthesis. Focusing on the multi-factor synthesis analysis method, Wang et al.^9^ selected seven influencing factors significantly related to the danger of highway debris flow, using pathway analysis, and evaluated the hazards of highway debris flows in the Daxigou area by combining the findings with the topology method. Huang Qile et al.^10^ proposed the automatic quantitative division of slope units as the basis of regional debris flow hazard evaluation, selected eight evaluation indexes, and established an APH-RBF neural network evaluation model to evaluate the debris flow hazard. Hairong Ma et al.^11^ used the hierarchical analysis method to assess the debris flow hazard in the Sego area and analyzed risk along the Sego Expressway. Chen Wei et al.^12^ used the fuzzy comprehensive judgment method to evaluate the danger of debris flow along a highway in northwest Yunnan. When the multi-factor comprehensive analysis method is used to quantify the factors, field investigations should also be undertaken, as these are more suitable for the specific risk assessment of debris flow events. Although these evaluation methods are simple and feasible, they fail to fully consider the correlation between evaluation factors and the degree of risk. In some cases, it is difficult to obtain all evaluation indicators. Using the geomorphic information entropy evaluation method, some scholars assessed the development stage and performed danger degree classification for debris flow gullies and valleys based on geomorphic evolution theory from the point of view of the area–height curve function and information entropy^13–17^. Liu Lina et al.^18^ evaluated the risk of debris flow in the Lushan earthquake area by combining the information entropy of the gully with that of the source of the landslides. Wang Jun et al.^19^ coupled gully information entropy and watershed unit material response rate to evaluate the debris flow hazard of the Magui River watershed in Gaizhou, western Guangdong. Xie Tao et al.^20^ evaluated the effects of debris flow in a glacier on the Tianshan highway using gully information entropy. A comprehensive multi-factor analysis method needs to be combined with field investigations to determine the value of the factors quantitatively, and this approach is more suitable for evaluating specific debris flow hazards^21^. On the contrary, evaluation methods using geomorphological information entropy can be based on topographic information data combined with remotely sensed imagery, which simplifies and optimizes the analysis of debris flow hazards via the powerful spatial analysis capability of GIS technology and is more efficient and accurate in evaluating debris flow hazards^22–25^. According to the information entropy value, the degree of development of geomorphic erosion and the geomorphic evolution stage of the basin can be identified. Subsequently, the risk caused by debris flow can be determined. In this process, the relationship between the location of the highway and the debris flow gully is not taken into consideration, so the accuracy of the evaluation results needs to be improved.

Geomorphic evolution theory takes the terrain conditions as a single factor when evaluating the risk of highway debris flow without considering the influence of loose material sources and water sources, and the evaluation results are biased to some extent. When the regional rainfall meets the critical rainfall for debris flow initiation, the development processes of debris flow are discussed with the help of geomorphic evolution theory, and the regional risk judgment matrix of debris flow is established by combining the information entropy of gullies and source sensitivity. Considering the location relationship between highway and debris flow gullies, a rapid evaluation method for debris flow risk assessment is proposed by modifying the judgment matrix. In this paper, four debris flow gullies along a section of the G318 (from Yajiang County to Jianziwan section) subjected to a quality improvement project are selected to verify the evaluation results. The appropriate engineering forms for a line crossing area affected by debris flow are analyzed, along with the proposal of an appropriate route selection scheme.

## Overview of the study area

### Topographic and geomorphologic conditions

**Figure 1:**
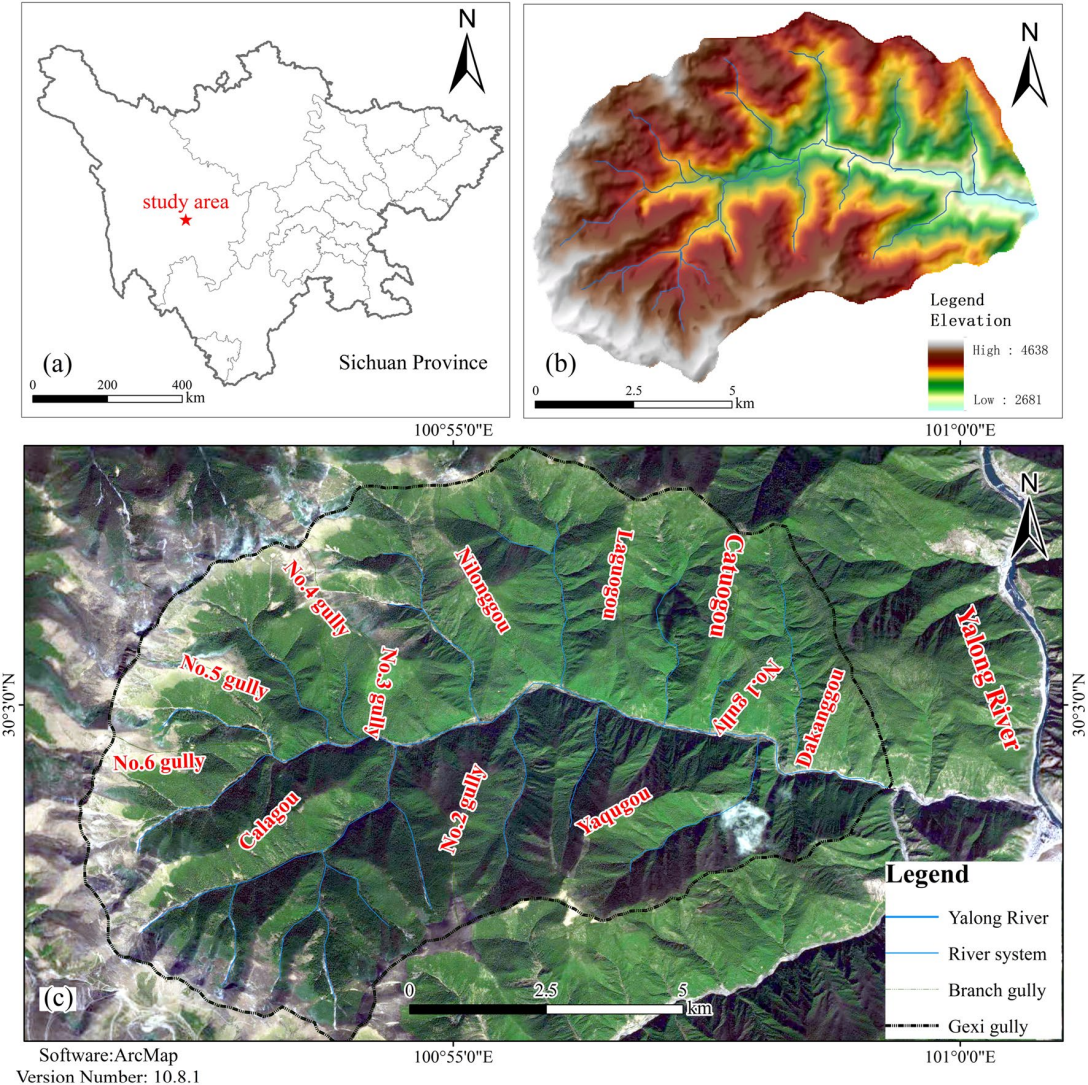
The overview map of the study area.

The judgeship watershed is located on the west side of Yajiang County, Ganzi Tibetan Autonomous Prefecture, Sichuan Province (Fig. 1a), with the first-level tributary on the left side of the Magogou and the second-level tributary on the right side of the Yalong River. Figure 1b illustrates the study area: the Gexi gully basin has an area of 76.74 km^2^; the main gully has a length of 10.41 km; the average longitudinal drop of the gully is about 284‰; both sides of the slope are steep, generally in the 15°–60°; the vegetation coverage rate is 60–80%; the highest point of the watershed’s elevation is 4600 m; the lowest point is the outlet of the gully, with an elevation of 2750 m; the maximum relative height difference is 1850 m. The morphology of the gully is a "balsam leaf shape". The watershed features many branching gullies, including three branched gullies on the right side of the main gully and nine on the left side (Fig. 1c). The area of the watershed is between 0.88 and 14.82 km^2^, the average slope is between 212 and 684‰, the longitudinal drop of the gully is significant, the maximum height difference in the watershed is more than 1000 m, and the average slope of the mountain is 30°–60°. Upstream of the gully, the high-altitude area shows the intense weathering of rocks and soils on the slopes, and more bedrock is exposed. Downstream of the gully, there are many avalanches and slides, with better conditions for the production and confluence of flash floods and debris flow, as well as better conditions for the formation of the topography and geomorphology required for debris flow.

The images in Fig. 1 were created using ArcMap software, version 10.8.1. Figure 1a represents data obtained from DaTaV GeoAtlas (https://datav.aliyun.com/portal/school/atlas/area_selector). The DEM Data in Fig. 1b is derived from the Geospatial Data Cloud (https://www.gscloud.cn/#page1/2) at a scale of 1:50,000. Figure 1c displays data from the BIGMAP GIS office (version 30.0.0.0; www.bigemap.com). The imaging was conducted on July 21, 2022, at a scale of 1:1953. The watershed in Fig. 1 is divided into sub-watershed units using spatial Analyst Tools in ArcToolbox within ArcMap. This process involved seven main steps, including using the Fill function of Hydrology to fill the low-lying land, the Flow Direction function of Hydrology to analyze the Flow direction, and the Flow Accumulation function of Hydrology to calculate the flow quantity. The river network was constructed using The Raster Calculator function in Map Algebra. The stream order function in hydrology optimized the raster data, and the watershed function extracted sub-watershed division.

**Figure 2:**
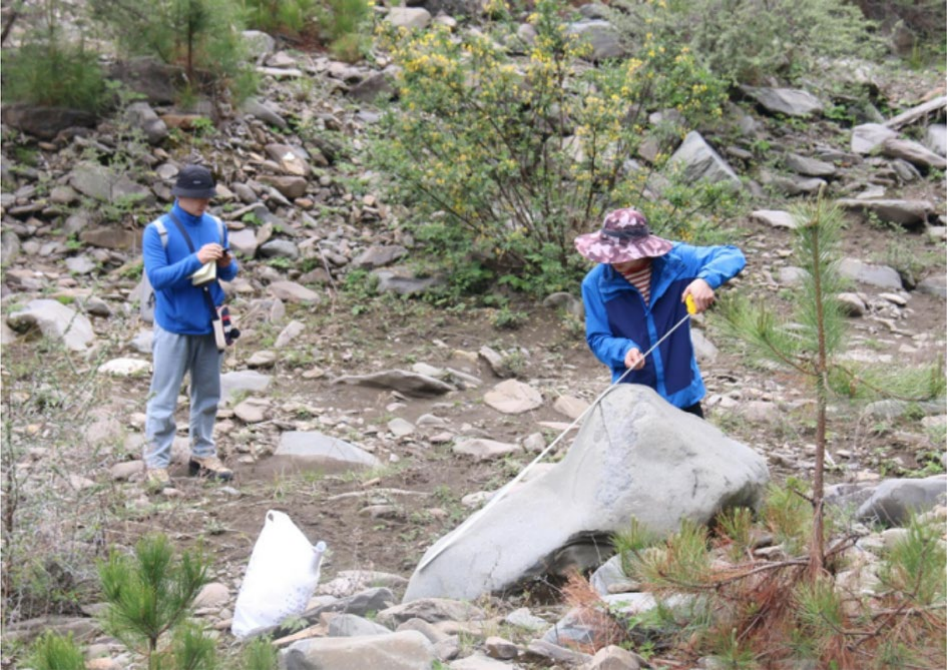
Ancient debris flow deposits in the gully of Gexigou watershed.

The field investigation showed that the grain sizes of the stone accumulations in the gully were within the range of 10–60 cm. The average grain size of the stones in the middle and lower reaches of the rocky beach was 1.2 m, and a relatively large amount of coarse driftwood was found in the gully (Fig. 2).

**Figure 3:**
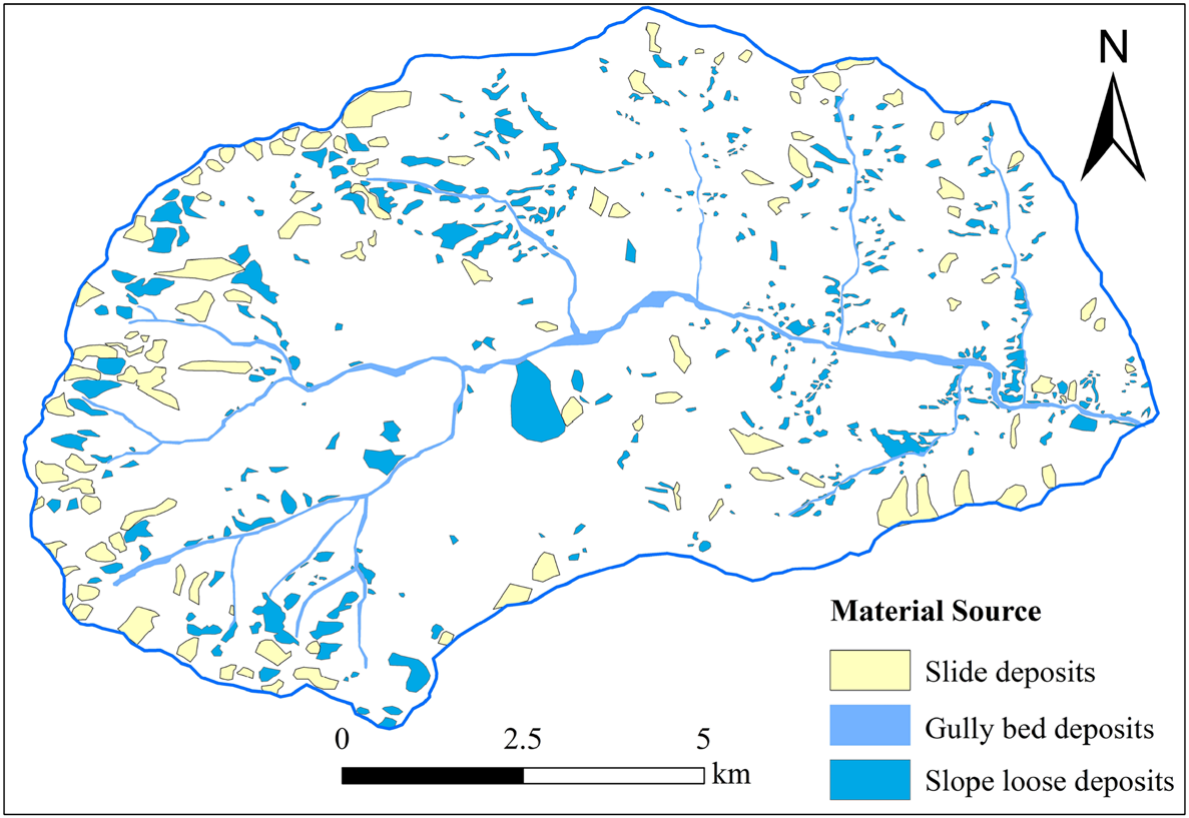
Types of loose solid materials and their distribution in the Gexigou watershed.

Based on the interpretation of remote sensing images, the types, distribution, and storage capacities of debris flow material sources in the Geshigou basin were identified and estimated. It was found that the material sources mainly included four types: landslide material sources, channel accumulation material sources, slope erosion material sources, and old debris flow accumulations (Fig. 3). The total amount of loose solid material sources in the Geshigou watershed was calculated to be about 6.714 × 10^8^ m^3^, and the amount of kinetic storage that could be involved in debris flow activities was 2.086 × 10^8^ m^3^. The magnitudes of the storage and recharge capacities of the abundant loose solids would have a critical impact on the scale and nature of debris flow hazards.

### Meteorological and hydrological conditions

The region is part of the tectonic denudation of a high mountainous terrain with significant elevation differences. In the mid-mountainous area, the elevations of 2500–4500 m are dominated by a few peaks greater than 4000 m. The directions of the mountains coincide with the tectonic line. The climate is of the continental monsoon plateau type, affected by the geographic location, topography, and regional climate. It is characterized by dry and wet seasons, with extended hours of sunshine and radiation. The average annual temperature is generally in the range of 4–10 °C, with the lowest being − 6.5 °C and the highest being 14.8 °C. Rainfall in the basin is relatively abundant, generally ranging from 1300 to 1600 mm, and in extreme cases, the annual rainfall is less than 100 mm, mainly concentrated from June to September; the rainfall accounts for 78% of the total annual rainfall. After October, the average monthly rainfall is reduced to the level seen in the dry season for half a year.

### Geological background conditions

**Figure 4:**
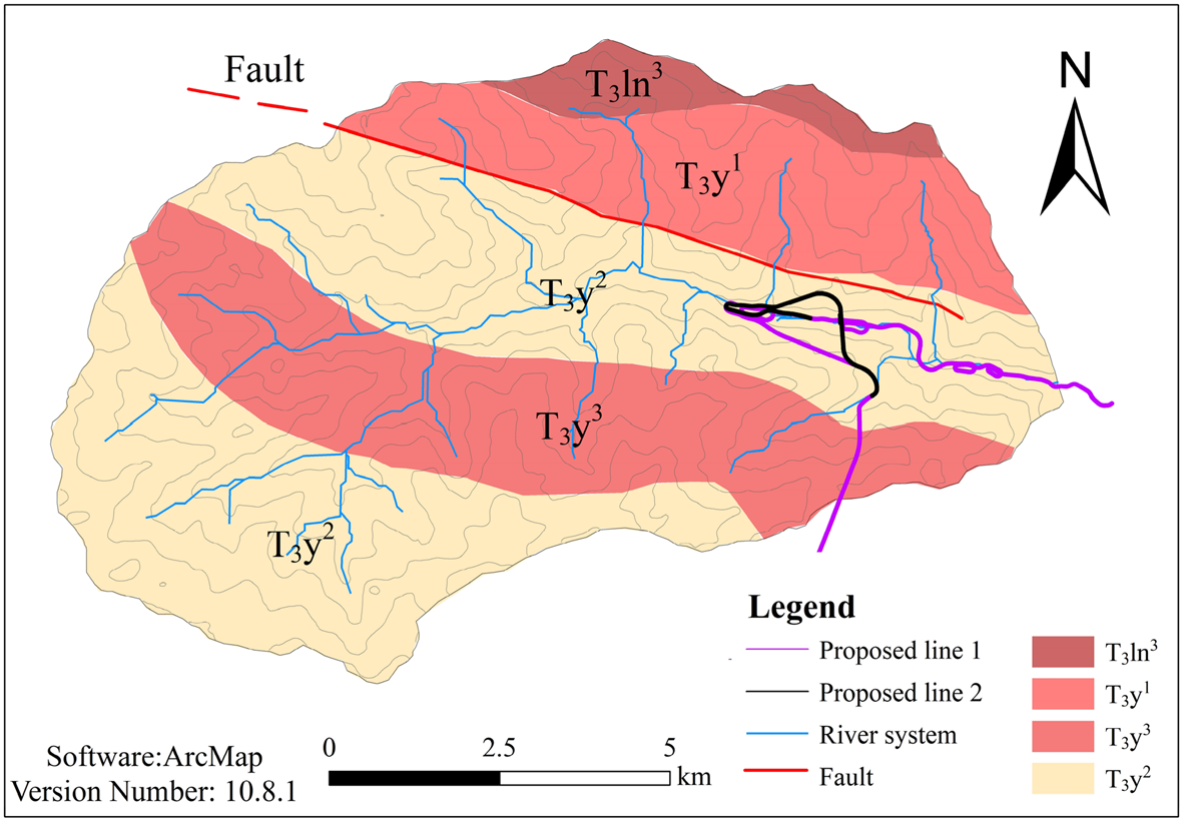
Engineering geological map of Gexigou watershed.

The study area is located on the Western Sichuan Plateau in the Sichuan Basin transition zone, stretching across the Yangzi land mass area and the Qiangtang–Sanjiang orogenic system of two major tectonic plates. Since the Quaternary period, under the influence of tectonic activity, the terrain has been consistently uplifted; ruptures have strengthened the formation of a series of rift valleys along the fracture zone depressions, and the two forms of weathering have reduced the significance of the handling. The study area shows intense tectonic activity and fault development. The basin features a reverse fault across its entire length, extending east–west. The downstream area is divided by this fault, and the exposed strata in the study area can be ranked in order from old to new as the upper Triassic Yajiang Formation, the upper section of the upper Triassic Lianghekou Formation, and the Quaternary (Q) accumulation layer (Fig. 4).

## A rapid evaluation method of highway debris flow hazard based on gully information entropy

The conditions that foster debris flow formation include steep topographic and energetic conditions, abundant physical resources determined mainly by geological conditions, and abundant water power conditions. Among these, the topographic conditions are the most fundamental and decisive conditions for the occurrence of debris flow. The topographic conditions necessary for debris flow development are usually indicate potential debris flow in any valley. On the contrary, the formation of debris flows is unlikely or even impossible if the topographic conditions are not present, no matter how rich the solid material is in the valley and how intense the rainfall is. In this sense, topographic conditions play a critical role in the formation of debris flow, which is an essential consideration when studying debris flow formation mechanisms^26^. Based on geomorphic evolution theory, rapid evaluations of highway debris flow hazards can be applied in studies of the feasibility of proposed routes by virtue of the easy access to data sources and the convenient and accurate evaluation process.

### Information entropy calculation method for gully

According to the theory of the geomorphology of debris flow erosion, Strahler's area–elevation analysis method can be used in combination with information entropy to quantitatively analyze the erosion of the watershed system^13,17,27^. The expression of this method is:

1 $$G = S - 1 - \ln S = \int_{0}^{1} {f\left( x \right)} dx - 1 - \ln \left[ {\int_{0}^{1} {f\left( x \right)dx} } \right]$$

where $G$ denotes gully information entropy, and $S$ denotes the value of Strahler’s area–height integral.

The area-elevation integral was proposed by Strahler, an American theoretical geomorphologist, in the 1950s to quantitatively analyze the developmental stages of watershed geomorphology^28–30^. The specific calculation method is as follows:

$A$ denotes the watershed area of the gully, $m^{2}$; $a$ denotes the area above the contour in the watershed, $m^{2}$; $h$ denotes the height difference between this contour and the lowest point in the watershed, $m$; $H$ denotes the maximum height difference in the watershed, $m$.Based on the measured data, a curve is fitted and drawn in the rectangular coordinate system, and the best function can be obtained. This function (2) is the area–elevation curve of the basin:

2 $$y = f\left( x \right)$$

here, $x = a/A,y = h/H$. For the area–elevation curve of the watershed, we must integrate over the area within the $X$ and $Y$ axes.

Debris flows are highly erosive, resulting in debris flow gullies with more intense erosive action than the average erosive watershed system. At the same time, the geomorphic characteristics of the watershed strongly influence the development, progression, and recession of debris flows. The relative height of the watershed determines the size of the potential energy of the flowing water, which provides kinetic energy for the initiation of loose debris material. The area of the watershed and the amount of precipitation it receives are the main parameters determining the hydrodynamic conditions of the gully valley, and they are also important factors affecting the occurrence of debris flow. Therefore, the quantitative calculation of the geomorphic erosive state of the gully valley can reflect the development stage of the debris flow to a certain extent and determine its danger^30^.

### Gully information entropy and debris flow development stage classification

Based on the information entropy of the gully, the development stage of a gully’s geomorphology can be divided into three stages: the juvenile stage $G > 0.111$, the prime-age stage ($0.111 \le G \le 0.4$), and the old-age stage ($G > 0.4$)^13,17^. However, some scholars further subdivide the gully developmental stage of prime-age into the prime young stage and the prime old stage, thus forming a quadratic division, comprising the juvenile stage ($G < 0.15$), the prime young stage ($0.15 \le G \le 0.4$), the prime old stage ($0.4 \le G \le 0.6$), and the prime old stage ($G > 0.6$)^16^. In this study, based on the information entropy of the gully and the developmental characteristics of the debris flow in the study area, referring to previous research results^31–33^, the development and evolution process of debris flow can be divided into five stages, which are the developmental stage, the development period, the prime period, the decline phase and the layoff period (Table 1).

**Table 1:** Division of formation and development stages of debris flows.

Geomorphic stage of gully development	Landform features of the watershed	Information entropy of the watershed ($G$ value)	Development stage of debris flow
Infancy	Gradual development of gullies, gradual development of gully debris flows, increasing rate of siltation, small scale	(0, 0.11)	Developmental stage
Adult (juvenile) period	Debris flows develop, gully slopes are unstable, siltation rate increases, large scale	[0.11, 0.20)	Development period
Adolescence	Extremely developed debris flows, highly unstable gully slopes, steady rate of siltation, large scale	[0.20, 0.30]	Prime period
Prime (old age) period	Debris flows gradually subside and are less easily occur, gully slopes tend to be stabilized, and riverbed erosion is dominant, with siltation and washout	[0.30, 0.40)	Decline phase
Old age	Debris flows are rare, gullies are stabilized, vegetation is restored, and a quasi-plain is gradually developed	≥ 0.4	Layoff period

### Data processing and analysis of results

**Figure 5:**
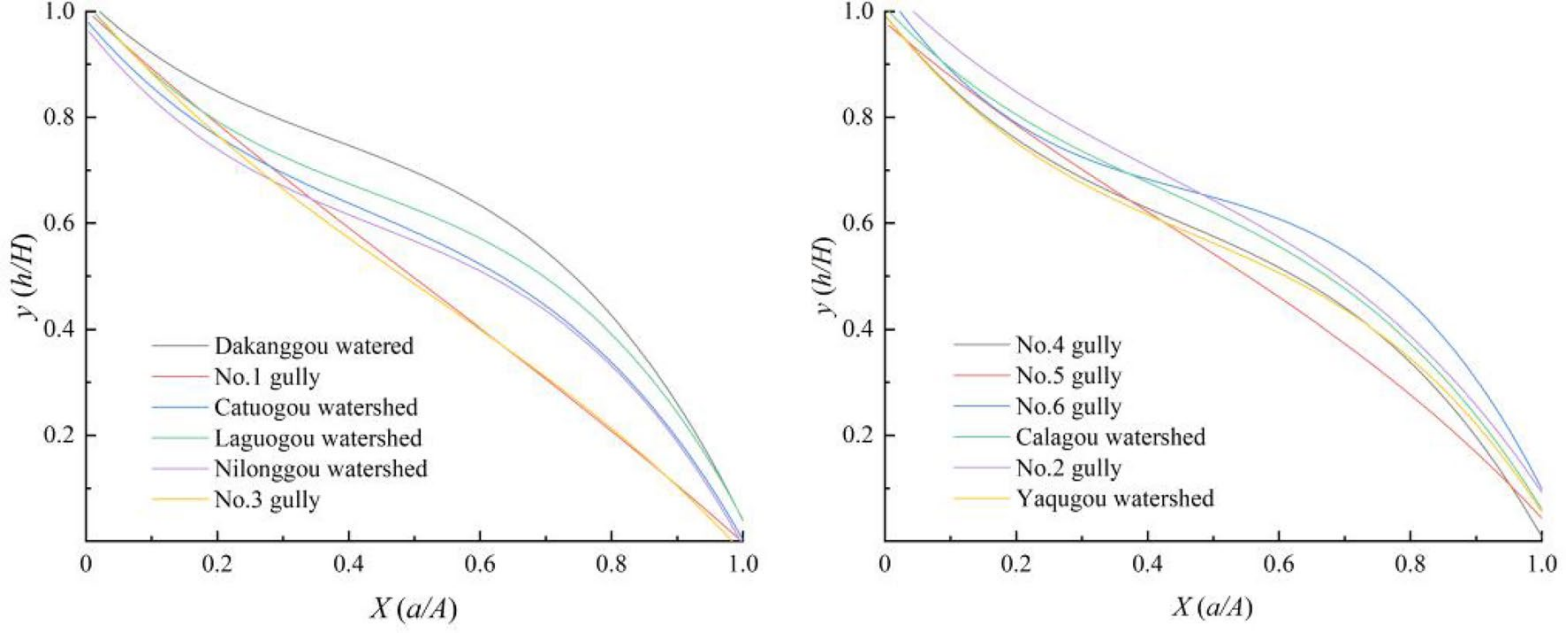
Area–Elevation Curve of Gexigou watershed.

Using the spatial analysis function of ArcGIS software in the 1:50,000 DEM, the watershed boundaries were divided into vectorized processes based on the topographic contour data of the study area. The watershed area corresponding to the contour distance of each gully was 50 m or more (corresponding to contour intervals), and the data source was converted into an EXCEL recognizable format. The obtained data were imported into EXCEL software to summarize and organize them for curve-fitting analysis, and the gully development area–height curve function was drawn for trend fitting. In this study, the EXCEL tool was used to compare the fitting effect using linear fitting, logarithmic equations, polynomial equations, and other curve models. The results show that the fitting effect of all curves was best when the polynomial equation was of the third order (Fig. 5) and the fitting degree of each gully curve, R^2^, was greater than 0.984, thus meeting the requirement of computational precision (the closer the fitting degree value is to 1, the greater the effect of the fit).

Using Matlab software, the geomorphic information entropy $G$ was obtained by integrating the area–height curve equation in the interval [0, 1] for each debris flow gully’s development (Table 2).

**Table 2:** Information entropy and debris flow development stages of all gullies in the study area.

Name of the gullies	$R^{2}$	Strahler area–elevation curve function *X* = [0,1]	$S$	$G$	Developmental stage	Risk classification
Dakanggou water	0.9960	$y = - 0.2448x^{3} + 0.3827x^{2} - 1.1433x + 1.003$	0.6417	0.0854	Developmental stage	High
No. 1 gully	0.9982	$y = - 0.2448x^{3} + 0.3827x^{2} - 1.1433x + 1.003$	0.4977	0.1954	Development period	Relatively high
Catuogou watershed	0.9944	$y = - 1.9179x^{3} + 2.4863x^{2} - 1.5452x + 1.0177$	0.5536	0.1449	Development period	Relatively high
Laguogou watershed	0.9973	$y = - 0.2448x^{3} + 0.3827x^{2} - 1.1433x + 1.003$	0.5944	0.1146	Development period	Relatively high
Nilonggou watershed	0.9985	${\text{y}} = { - 1}{\text{.8524x}}^{{3}} + 2.4372{\text{x}}^{{2}} - 1.5625x + 0.9696$	0.5376	0.1582	Development period	Relatively high
No. 3 gully	0.9962	${\text{y}} = { - 0}{\text{.7417x}}^{{3}} + 1.1649{\text{x}}^{{2}} - 1.4645x + 1.0189$	0.4895	0.2038	Prime period	Medium
No. 4 gully	0.9995	${\text{y}} = { - 1}{\text{.7721x}}^{{3}} + 2.361{\text{x}}^{{2}} - 1.5745x + 0.9936$	0.5503	0.1476	Development period	Relatively high
No. 5 gully	0.9960	${\text{y}} = { - 1}{\text{.621x}}^{{3}} + 2.2779{\text{x}}^{{2}} - 1.5951x + 0.9931$	0.5320	0.1631	Development period	Relatively high
No. 6 gully	0.9847	${\text{y}} = { - 2}{\text{.3569x}}^{{3}} + 3.2161{\text{x}}^{{2}} - 1.8005x + 1.0395$	0.6221	0.0968	Development period	High
Calagou watershed	0.9957	${\text{y}} = { - 1}{\text{.4182x}}^{{3}} + 1.7831{\text{x}}^{{2}} - 1.3123x + 1.0077$	0.5914	0.1167	Development period	Relatively high
No. 2 gully	0.9879	${\text{y}} = { - 1}{\text{.198x}}^{{3}} + 1.5128{\text{x}}^{{2}} - 1.2754x + 1.0525$	0.6196	0.0983	Developmental stage	High
Yaqugou watershed	0.9971	${\text{y}} = - {1}{\text{.621x}}^{{3}} + 2.2779{\text{x}}^{{2}} - 1.5951x + 0.9931$	0.5496	0.1482	Development period	Relatively high

**Figure 6:**
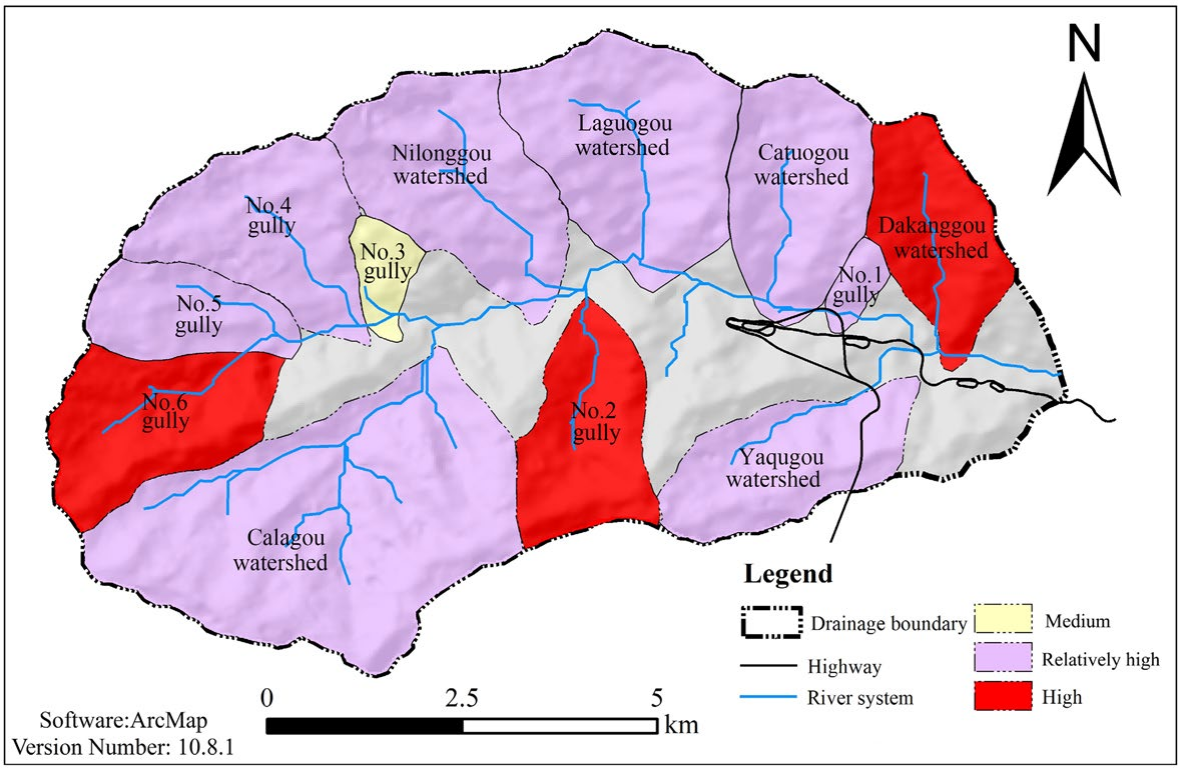
Results of debris flow risk assessment in each branch of the gullies (evaluation of debris flow risk on the basis of topographic and geomorphologic conditions).

With reference to Table 1, the debris flow hazard zoning map is shown in Fig. 6.

## Rapid evaluation method of highway debris flow hazard based on material response rate of watershed unit

### Methodology for calculating material response rates for watershed units

The response rate of slope unit mass movement (RRSUMM) is the average distribution area of loose material in a watershed unit, proposed by Wang Jun^19^. It measures the degree of aggregation of loose material sources in a watershed unit, and a larger value in this index indicates that the loose material sources in the watershed unit are more abundant, which is taken as the basis to analyze the sensitivity of the source of the gully grain. The expression is as follows:

3 $$RRSUMM = \frac{S}{A}$$

In the formula, $RRSUMM$ denotes the watershed unit material response rate, dimensionless; $S$ denotes the area of loose solids within the watershed unit, km^2^; $A$ denotes the watershed unit area, km^2^.

### Data processing and analysis of results

**Figure 7:**
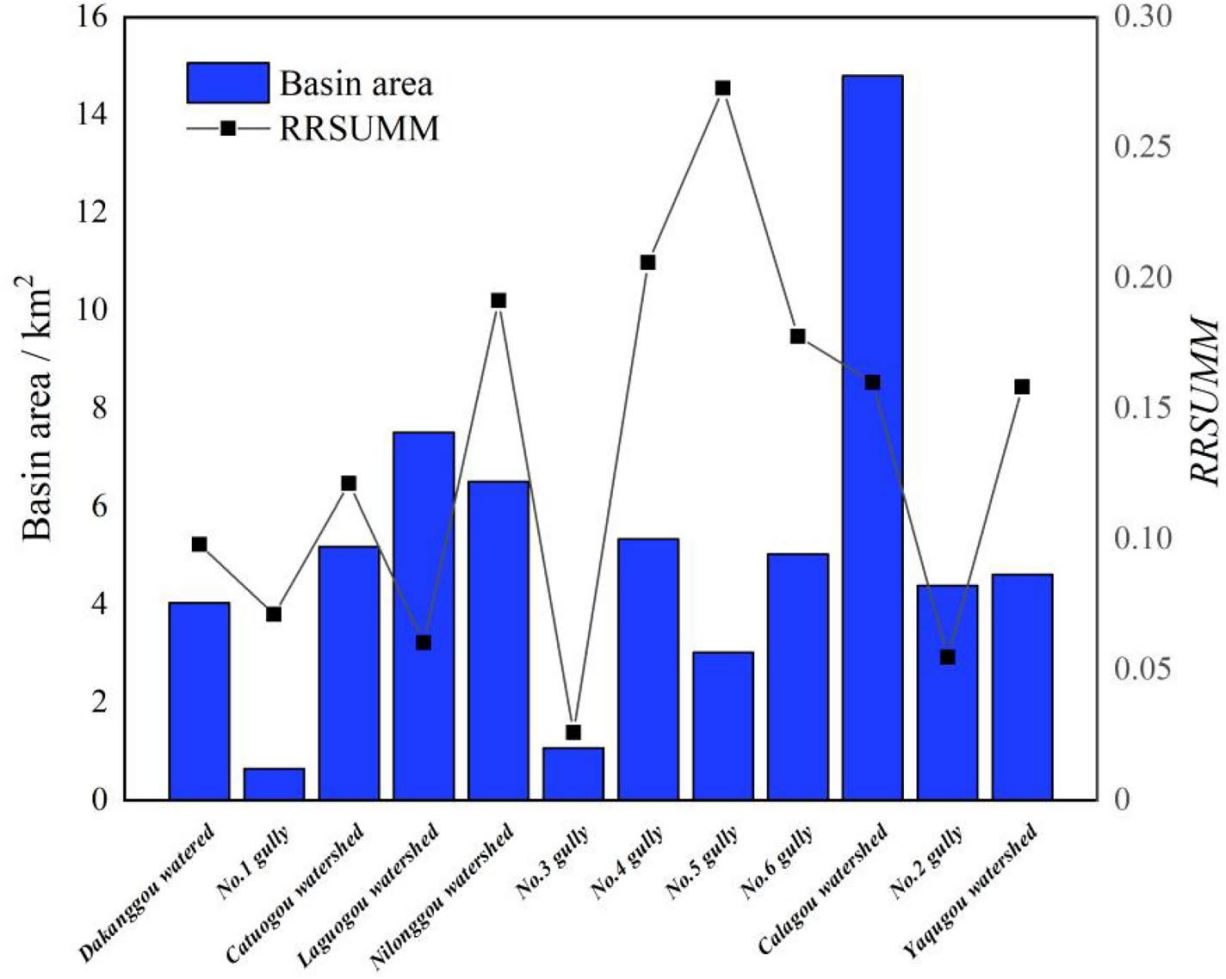
Slope response rate curve of each of the branching gullies.

Figure 3 shows the spatial distribution of loose material sources in the study area. The area of loose material sources in each sub-basin was counted using the spatial analysis function of ArcGIS software to obtain the unit material response rate of each sub-basin $RRSUMM$ (Fig. 7).

In the study area, RRSUMM ranges from 0.026050 to 0.272754. ArcGIS was used as the operating platform to classify the object source sensitivity into five levels using the natural intermittent classification method. These levels include the highly sensitive area, the relatively highly sensitive area, the medium sensitivity area, the lower sensitivity area, and the insensitive area, as shown in Table 3.

**Table 3:** Sensitivity statistical analysis table of the loose solid materials (based on the sensitivity of the loose solid materials).

$0.206032 \le RRSUMM \le 0.272754$(be) worth	Sensitivity of the loose solid materials
$0.206032 \le RRSUMM \le 0.272754$	Highly sensitive
$0.206032 \le RRSUMM \le 0.272754$	Relatively highly sensitive
$0.071230 \le RRSUMM \le 0.121396$	Medium sensitivity
$0.026051 \le RRSUMM \le 0.071229$	Low sensitivity
$0 < RRSUMM \le 0.026051$	Insensitive

**Figure 8:**
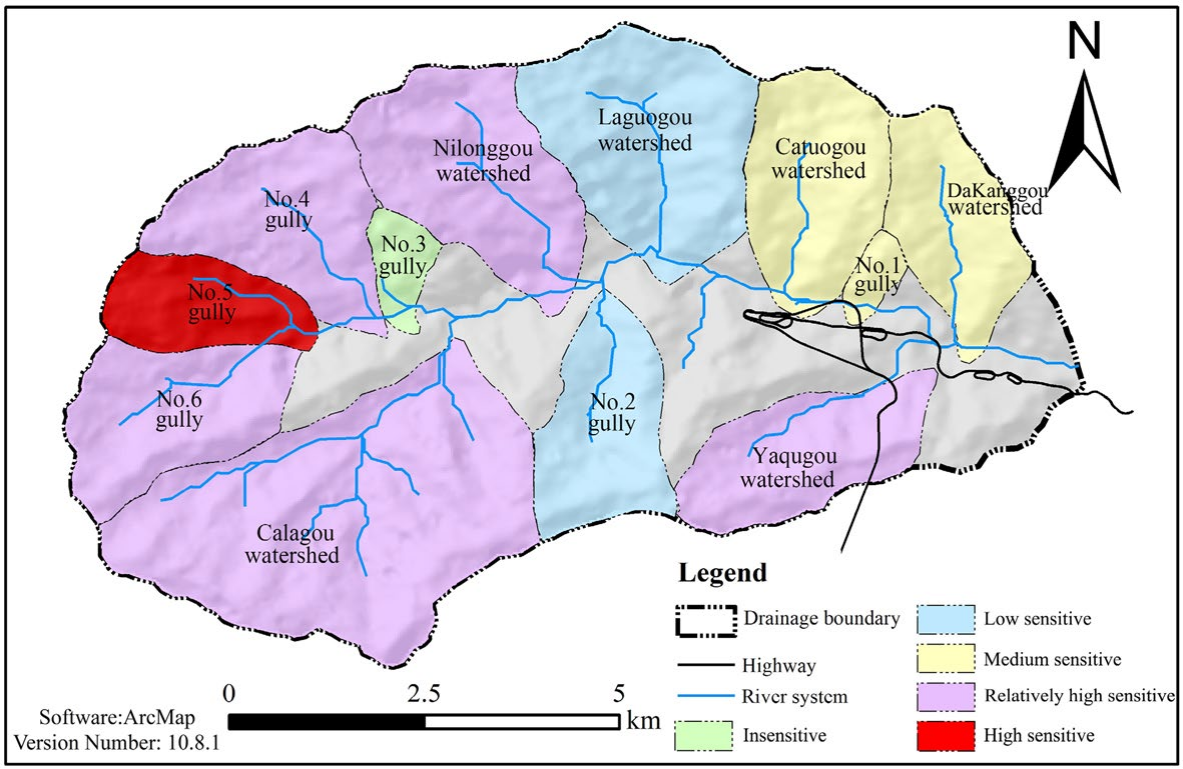
Sensitivity distribution map of the loose solid materials in each gully.

With reference to Table 3, a risk zoning map of the debris flows is shown in Fig. 8.

## Hazard analysis of highway debris flow under the coupling effect of terrain and loose solid materials

### Fundamentals

The multi-year average rainfall in the watershed is 652 mm, with the maximum daily rainfall of 602 mm. The maximum 10-min rainfall average falls between 5 and 7.5 mm, and the maximum 1-h rainfall average is 12.5 mm. Considering these rainfall statistics, the area is prone to debris flow outbreaks. When assessing debris flow hazards, it's crucial to consider both topographical and physical factors to ensure the accuracy of the results, as these factors are interlinked. Due to the inconsistencies in the data source and magnitude, the gully information entropy $G$ value and unit material response rate $RRSUMM$ are normalized and dimensionless, and their expressions are as follows:

4 $$X^{ * } = \frac{{X - X_{\min } }}{{x_{\max } - x_{\min } }}$$

$X^{ * }$ denotes the sample-normalized value; $X$ denotes the sample value; $X_{\min }$ denotes the sample data minimum value, and $x_{\max }$ denotes the sample data maximum value.

The smaller the value of the material response rate, the sparser the material sources in the watershed unit, and the lower the risk of debris flow; the smaller the value of the gully’s information entropy, the more severe the erosional wear of the watershed; the more intense the tectonic movements, the more active the debris flow and the higher the risk^19^. The unit material response rate $RRSUMM$ is taken as a negative value and then superimposed with the information entropy, which is the value of D. The value of D ranges approximately from − 0.5179 to 0.999. The classification method of natural discontinuity is used to classify the danger into five levels: high-danger area, relatively high-danger area, medium-danger area, relatively lower-danger area, and low-danger area.

### Data processing and analysis of results

ArcGIS software was used as a platform for debris flow risk classification. Table 4 presents the statistical partitioning of debris flow danger in each region.

**Table 4:** Hazard classification of debris flow under the coupling effect of terrain and loose solid materials.

Value of D	Debris flow risk classification
$- 0.5179 \, \le D \, \le \, 0.999$	High
$- \, 0.517912 \le D \le - \, 0.204203$	Relatively high
$- \, 0.204202 \le D \le - \, 0.005598$	Medium
$- 0.005597 \le D \le 0.116055$	Relatively low
$0.116056 \le D \le 0.99900$	Low

**Figure 9:**
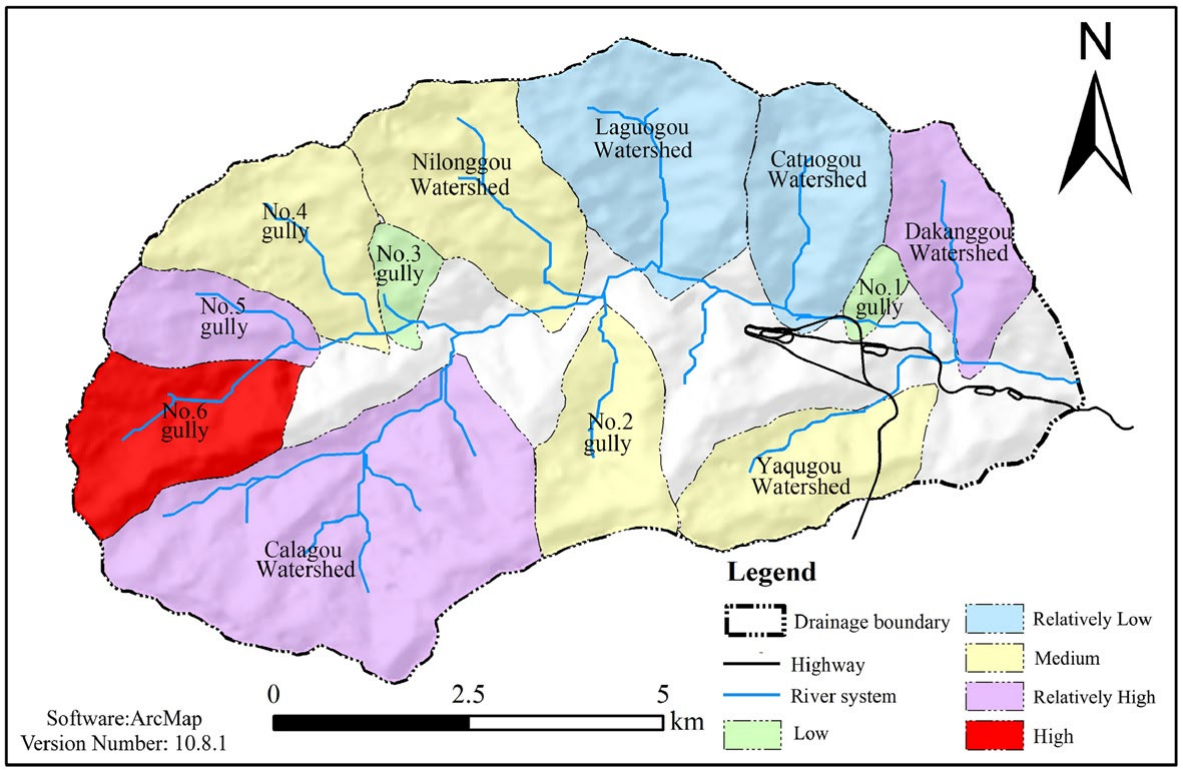
Hazard zoning map of debris flow under the coupling effect of terrain and loose solid materials.

With reference to Table 4, a comprehensive debris flow risk zoning map is shown in Fig. 9. From the figure, it can be seen that the wipe–pull gully is part of the higher-risk area. The evidence collected from the field features debris flow flood marks, supported by testimony from the local villagers, which contradicts the description given, indicating that the area being subjected to debris flow danger zoning features greater rationality than is given when the methods of the information entropy of the valley and the slope unit of the material response rate are used. This also accurately reflects the current and future trends of debris flow gully danger, which can be used to guide local disaster prevention and economic planning.

## Crossing program analysis

### Crossing program

**Figure 10:**
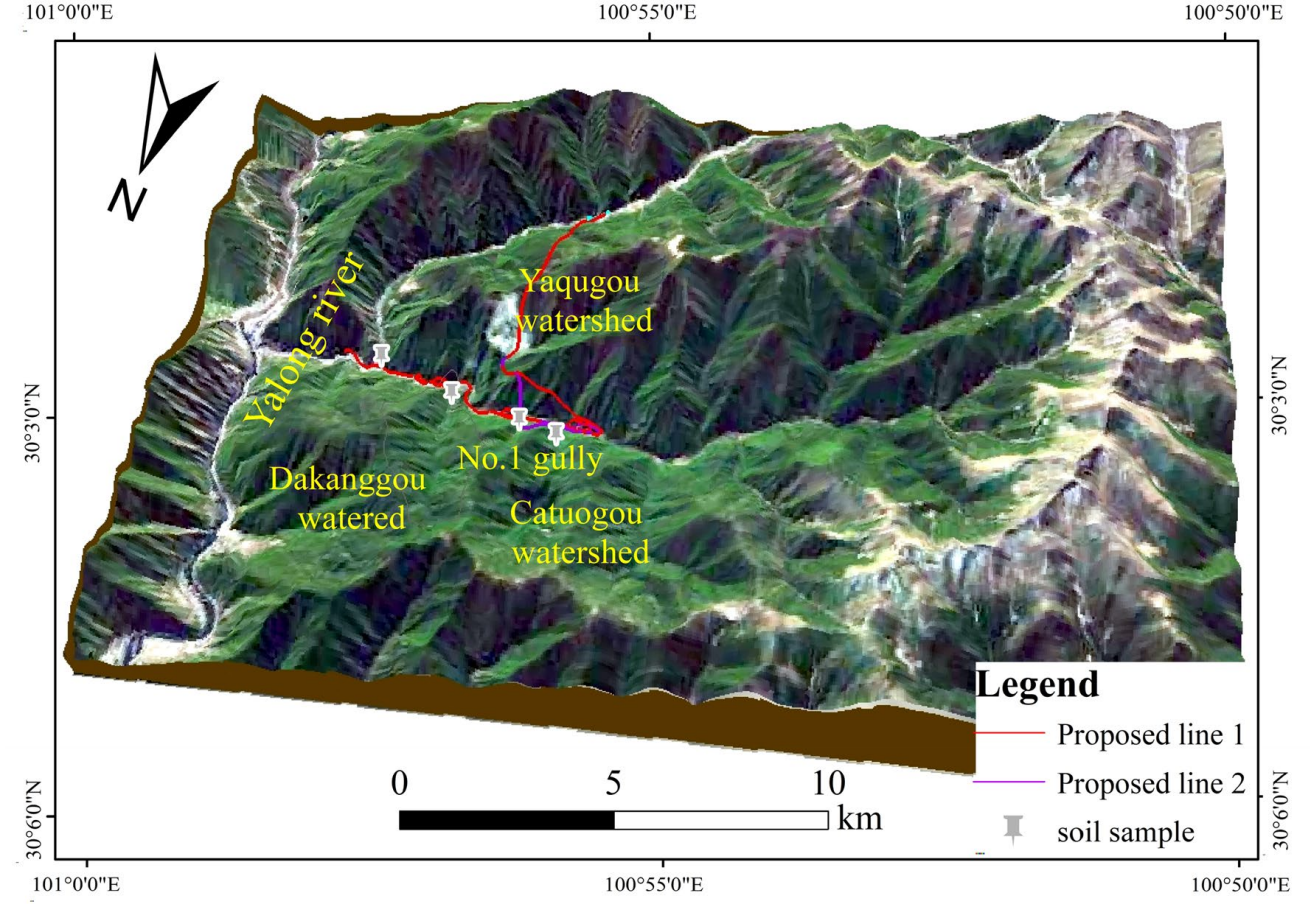
Location of the proposed highway and debris flow gullies.

The route starts from the intersection of G318 and Geshigou in Yajiang County, spreading along the Geshigou; it uses spiral bridges to spread the line uphill and tunnels to cross the mountains between Geshigou and Magogou and then connects back to the G318 near Magozong, with a total route length of 14.1 km. The traffic trunk line passes from the middle and lower reaches of the watershed. It passes through the debris flow accumulation area of the tributary gullies Dakang gully, 1# gully, and Weto gully via bridges before eventually crossing the Yaji gully (Fig. 10). Based on the coupling of the gully information entropy and slope unit material response rate methods, the hazard levels of Dakang gully, 1# gully, Wutuo gully, and Yazhou gully were determined to be high, low, low, and moderate, respectively. The effects change as the highway passes through debris flow gullies in different areas. Using a single debris flow gully as the center of the circle delineating highway hazards is obviously inappropriate. Instead, using the relationship between the locations of the highway and the debris flow to figure out the correct risk adjustment is more scientific.

### Evaluation of the degree of debris flow development

The evaluation of the degree of debris flow development is based on a comprehensive judgment of the danger posed by a debris flow gully and the scale of the debris flow that actually occurs. According to the volume of debris that potentially accumulates after being washed out following debris flow eruption, the scale of the debris flow eruption can be divided into four categories: extra-large debris flow volume is greater than or equal to 10^6^ m^3^; large debris flow volume is between 10^5^ and 10^6^ m^3^; medium-sized debris flow volume is between 10^4^ and 10^5^ m^3^; small debris flow volume is less than 10^4^ m^3^. The larger the scale of debris flow eruption, the stronger the degree of the corresponding debris flow that emerges in the developmental stage of the gully. A scale for grading the debris flow development degree was established according to the debris flow’s development stage and the size of the outburst (Table 5).

**Table 5:** Classification of debris flow development degree.

Risk of debris flow	Classification of debris flow development degree based on total solid materials		
	Giant debris flow or large debris flow	Medium-scale debris flow	Small-scale debris flow
Medium risk or relatively high risk	Strong development	Strong development	Moderate development
High risk	Strong development	Moderate development	Moderate development
Low risk or relatively low risk	Moderate development	Weak development	Weak development

### Classification of road debris flow hazards

The relationship between the location of the highway and the debris flow gully area was used to evaluate debris flow disasters on mountain roads. The vital indicators for the valley debris flow area can be generally divided into the formation, circulation, and accumulation areas. The impacts of each area on the highway show corresponding differences. The formation area is the starting point of a debris flow, and the highway’s foundation in this area is prone to local washout. Highways running through the debris flow gully accumulation area can be easily buried up to the pavement, causing the erosion of the roadbed and the burial of culverts. The foundation of a highway running through the circulation area is prone to washout, resulting in highway fracturing, which means the highway cannot be traversed^33,34^. The foundation of a highway running through the flow area is easily washed away, resulting in the highway being broken and becoming impassable. This study combines the positional relationship between highways and debris flows with the degree of development of debris flows, along with experience from previous research^35^. Thus, the danger posed by highway crossing debris flow was divided into three risk grades: large, medium, and small (Table 6).

**Table 6:** Debris flow risk classification (based on the scale of debris flow and the location between debris flow and highway).

Location relationship between highway and watersheds	Hazard classification		
	Strong development	Moderate development	Weak development
The highway passes through the transportation zone of the debris flow	High risk	High risk	Medium risk
The highway passes through the source area or depositional area of the debris flow	High risk	Medium risk	Weak risk
The highway is located outside the debris flow-affected area	Medium risk	Weak risk	Weak risk

### Evaluation results and validation

According to the above steps of the rapid evaluation of highway debris flow hazards, the parameters of highways passing through four debris flow gullies were obtained (Table 7). In order to verify the reliability of the above results, hazard evaluations of the four debris flow gullies, named Dakang, No.1, Wutou, and Yazhou, along the highway were performed by combining aerial photographs taken by drone with on-site investigations. The evaluation showed that the slopes on both sides of all gullies were unstable. The fans of accumulation at the mouths of the gullies were expanding, the central axes of the fronts of the debris flows were squeezed and shifted, and the vegetation cover was medium. There were many debris accumulations in river channels, and many impacted blocks of rock could be seen at the mouths of the gullies. Thus, the frequency of debris flow outbreaks was high. The proposed highway passes through the accumulation and circulation areas of debris flow gullies; it will feature a bridge to cross the flooding section, with a diversion wall around the bridge abutment to preclude the impacts of debris flow and regular cleans-up of debris flow accumulations around the diversion wall are planned.

**Table 7:** Risk assessment results of debris flow Gexigou watershed.

Name of the gully	Scale of debris flow	Development degree of debris flow	Location relationship between highways and watersheds	Hazard classification of debris flow
Dakanggou watershed	Small-scale debris flow	Moderate development	The highway passes through the debris flow depositional area	Medium risk
No. 1 gully	Small-scale debris flow	Moderate development	The highway passes through the debris flow depositional area	Medium risk
Catuogou watershed	Medium-scale debris flow	Strong development	The highway passes through the debris flow depositional area	High risk
Yaqu gou watershed	Medium-scale debris flow	Strong development	The highway passes through the debris flow transportation area	High risk

## Quantitative evaluation of debris flow hazards

### Evaluation of the maximum extent of impact of a debris flow

**Figure 11:**
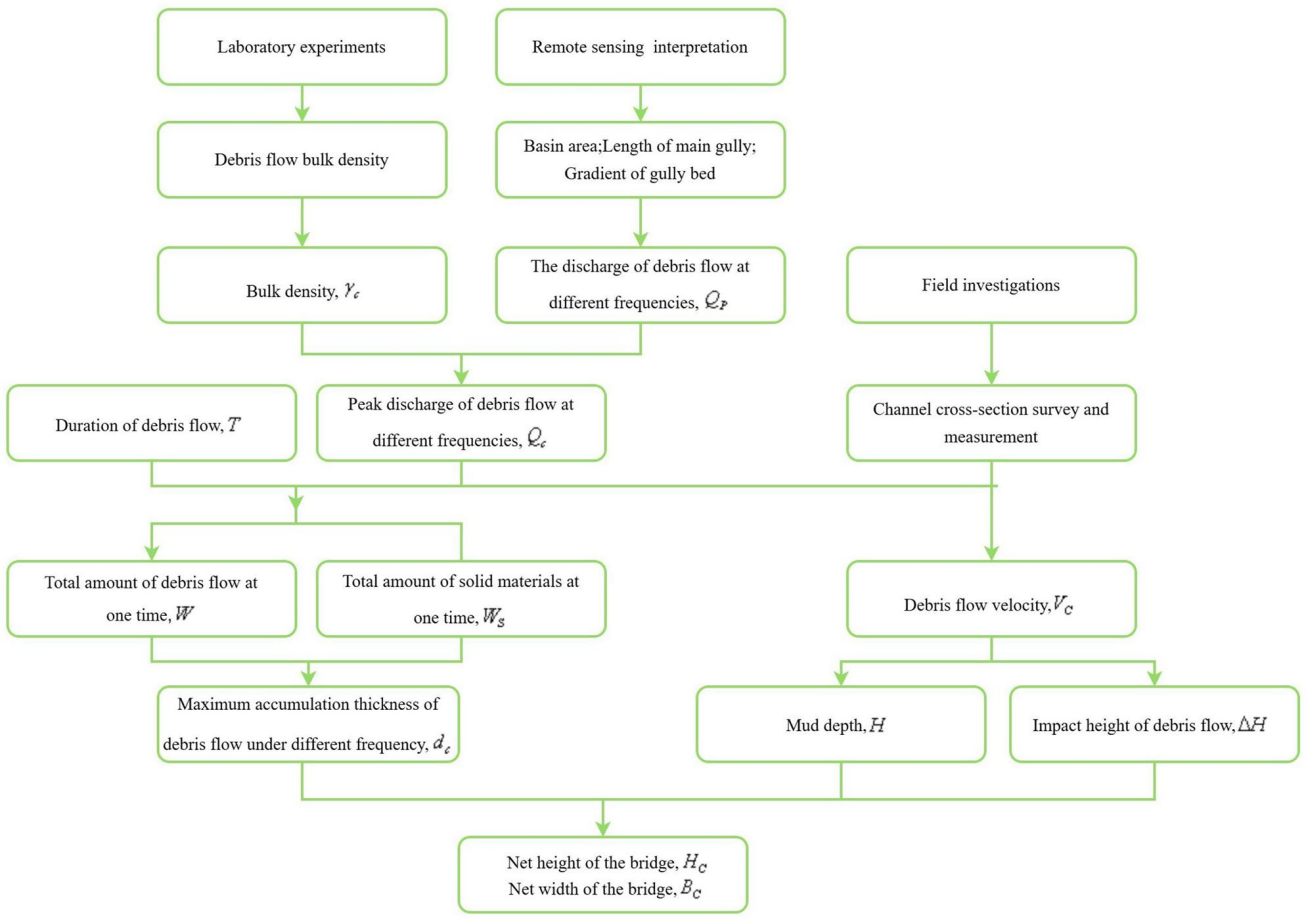
Quantitative evaluation of Debris flow hazards technical roadmap.

The degree of danger for the highway passing through Dakang gully and 1# gully is medium. In contrast, the danger associated with the highway passing through Ertuo gully and Yazhou gully is high. For a bridge passing through an area affected by debris flow, the bridge’s location, clearance height, and span are critical factors to consider. It is very important to evaluate the maximum possible impact area of a debris flow. According to the development characteristics of a debris flow, the maximum accumulation thickness, mud depth, and height of a debris flow all affect the extent of the impact area. The technical roadmap is shown in Fig. 11.

#### Peak discharge of the debris flow at different frequencies

The maximum siltation thickness of a debris flow at different frequencies was determined by calculating the peak debris flow flow. Due to the lack of meteorological and hydrological data for the Gexi gully and its tributary basins, it was impossible to use mathematical and statistical calculation methods to assess the peak flow of heavy rainfall. The China Academy of Water Resources Institute of Hydrology applied the "small watershed storm flood flow calculation method". This method applies to robust and unmeasured hydrological data and has a reasonable degree of practicality^36^. The formula is as follows:

5 $$Q_{p} = 0.278\psi SF/\tau^{n}$$

where $\tau^{n} = f\left( {m,s,I,L} \right);\psi = \left( {\mu ,\tau^{n} } \right)$; $Q_{P}$ denotes the design flow rate of a storm flood with frequency P, $m^{3} /s$; $\psi$ denotes the flood runoff coefficient; $S$ denotes the storm rain force for 1 h storm intensity, mm/h; $n$ denotes the storm rainfall index; $F$ denotes the watershed area, km^2^; $\tau$ denotes catchment time, h; $\mu$ denotes infiltration intensity, mm/h; $m$ is the confluence parameter.

For calculating heavy rainfall-induced storm-type debris flows, the rain–flood method is the most widely used. The formula for the rain–flood method is as follows:

6 $$Q_{c} = (1 + \varphi )Q_{p} \cdot D_{c}$$

where $Q_{c}$ denotes the peak discharge of debris flow for frequency P, m^3^ /s; $Q_{p}$ denotes the storm flood design flow rate for frequency P, m^3^/s; $\varphi$ denotes the sediment correction coefficient of debris flow, where $\varphi = {{\left( {\gamma_{c} - \gamma_{w} } \right)} \mathord{\left/ {\vphantom {{\left( {\gamma_{c} - \gamma_{w} } \right)} {\left( {\gamma_{s} - \gamma_{w} } \right)}}} \right. \kern-0pt} {\left( {\gamma_{s} - \gamma_{w} } \right)}}$; $\gamma_{c}$ denotes unit weight of debris flow, g/cm^3^; $\gamma_{w}$ denotes unit weight of clear water, g/cm^3^; $\gamma_{s}$ denotes unit weight of solid materials in the debris flow, g/cm^3^; $D_{c}$ denotes debris flow clogging coefficient.

**Figure 12:**
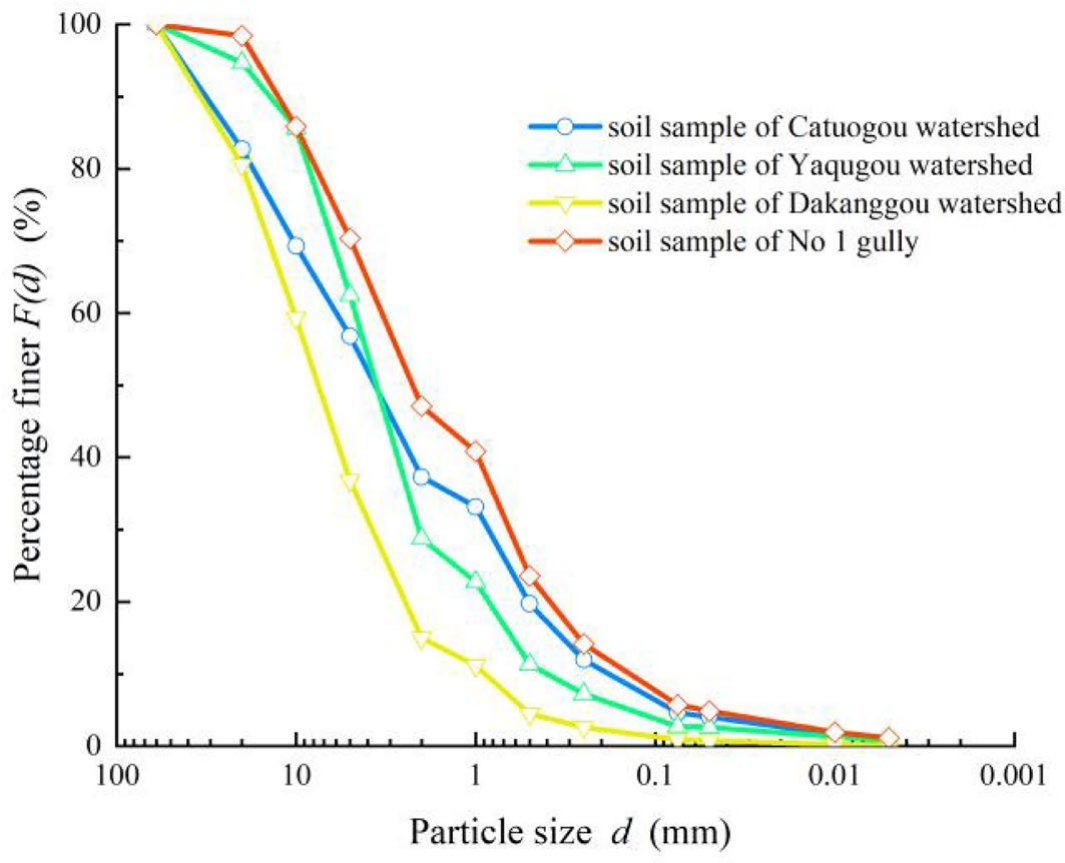
Particle size distribution curves of the soil in each gully.

Since there was no observed or recorded information available regarding the debris flow in Geshe gully, samples were collected from the debris flow accumulation fan (Fig. 10), and the particle screening and Marvin tests were used to analyze the material composition, particle gradation, and clay content of the debris flow. The calculation of the debris flow bulk density was performed using the debris flow bulk density calculation method based on clay content^37^, and the table-checking method was used to determine the capacity of the debris flow in the gully.

7 $$\begin{gathered} \gamma_{c} = - 1.32 \times 10^{3} x^{7} - 5.13 \times 10^{2} x^{6} + 8.91 \times 10^{2} x^{5} - 55x^{4} \hfill \\ \begin{array}{*{20}c}  &  \\ \end{array} + 34.6x^{3} - 67x^{2} + 12.5x + 1.55 \hfill \\ \end{gathered}$$

where $x$ denotes the content of clay particles in debris flow deposits with particle sizes less than 0.05 mm (Fig. 12).

#### Total amount flushed out by a single debris flow

The calculation of the total quantity of debris flow has been a challenging issue worldwide, and there are currently no scientifically reliable calculation methods available to estimate it. For this reason, the total content of one debris flow is empirically generalized to an ideal pentagon by the debris flow process line^38^. The formula is as follows:

8 $$W = 0.264Q_{C} \cdot T$$

where $W$ denotes the total amount of debris flow at one time, $m^{3}$; $T$ denotes the debris flow duration, $s$; the rest of the values hold the same meaning as described above.

The total amount of solids washed out by a debris flow, $W_{S}$, is determined according to the Specification for Investigation of debris flow Disaster Prevention and Control Engineering (from now on referred to as the "Specification")^39,40^. The formula is as follows:

9 $$W_{S} = W(\gamma_{C} - \gamma_{W} )(\gamma_{S} - \gamma_{W} )$$

where *W* denotes the total amount of debris flow at one time, m^3^; the rest of the values take the meanings given above.

#### Maximum accumulation thickness of a single debris flow

Among the several critical parameters of debris flow hazard assessment and prevention, the maximum accumulation thickness of a debris flow most directly affects the bridge clearance height. Due to the lack of yield stress data on debris flow in this region, a model for the prediction of the primary debris flow hazard range was used to determine its thickness, as proposed by Liu Xilin et al.^38^. The formula is as follows:

10 $$d_{c} = 0.017\left[ {\left( {V \cdot \gamma_{C} } \right)/(G^{2} \cdot \ln \gamma_{C} )} \right]^{\frac{1}{3}}$$

where $d_{c}$ indicates the maximum accumulation thickness of the debris flow, $m$; $V$ denotes the maximum recharge of loose solids, $m^{3}$; $G$ denotes the specific drop in the pile fall area; the rest of the meanings are the same as those described above.

#### Debris flow mud depth, maximum height of wash up

For the determination of debris flow velocity, as well as the mud depth and wash-up height, based on accumulation depth, the peak flow rate of debris flow at the cross-section is calculated as follows:

11 $$Q_{C} = V_{C} \cdot A$$

where $V_{C}$ indicates the debris flow velocity, m/s; $A$ denotes the debris flow overflow area, m^2^.

In the "Specification", the mud depth is determined by calculating debris flow velocity, with the application of the empirical formula for the region. The formula is as follows:

12 $$V_{C} = \frac{1}{n}H^{\frac{1}{3}} I^{\frac{1}{2}}$$

where $n$ denotes the roughness; $H$ denotes mud depth, m; $I$ denotes the cross-section of the specific drop.

In the absence of on-site mud samples, the maximum uplift height of a debris flow is calculated in accordance with the "normative" formula. The formula is as follows:

13 $$\Delta H = V_{C}^{2} /(2g)$$

where $\Delta H$ denotes the height of the punch-up, m; *g* denotes the acceleration of gravity, m/s^2^.

According to the above calculation method, Table 8 shows the results of the motion characteristic parameters of the four debris flow gullies in the region.

**Table 8:** Calculation results of dynamic parameters of the debris flow in the Gexigou watershed.

Name of the cross-section	Cross-section location	$P_{}$/%	$\gamma_{C}$ (g/m^3^)	$Q_{p}$ (m^3^/s)	$Q_{C}$ (m^3^/s)	$W$ (10^4^m^3^)	$W_{S}$ (10^4^m^3^)	$V_{C}$ (m/s)	(m)	$d_{c}$ (m)	$\Delta H_{}$ (m)
Cross-section of Daganggou watershed	N 30° 2′ 29.23″ E 100° 58′ 26.71″ H: 2793 m	1	1.74	8.55	46.53	0.74	0.33	3.16	1.7	1.37	0.51
		2	1.63	7.91	35.81	0.51	0.19	2.46			
		5	1.55	6.32	26.53	0.34	0.11	2.28			
		10	1.46	5.01	19.45	0.22	0.06	1.79			
Cross-section of No. 1 gully	N 30° 03′ 00.78″ E 100° 57′ 30.07″ H: 2950 m	1	1.67	6.89	17.40	0.28	0.11	4.80	1.2	1.54	0.85
		2	1.59	6.21	14.44	0.21	0.07	4.56			
		5	1.47	5.30	11.15	0.14	0.04	4.32			
		10	1.39	4.55	8.93	0.10	0.02	4.25			
Cross-section of Yaqugou watershed	N 30° 02′ 53.41″ E 100° 57′ 57.45″ H: 2877 m	1	1.79	9.80	56.38	0.89	0.43	4.64	2.8	1.46	1.10
		2	1.68	8.46	40.31	0.57	0.23	3.84			
		5	1.60	6.37	29.63	0.38	0.13	2.80			
		10	1.51	5.31	21.52	0.24	0.07	2.59			
Cross-section of Catuogou watershed	N 30° 03′ 06.95″ E 100° 57′ 03.28″ H: 3002 m	1	1.79	11.15	64.19	1.70	0.93	4.35	2.7	1.63	0.97
		2	1.68	9.65	45.96	1.10	0.53	3.61			
		5	1.60	7.70	33.88	0.70	0.29	3.21			
		10	1.51	6.09	24.70	0.45	0.16	2.63			

### Line hazards and prevention countermeasures

In bridge design, the impact force of a debris flow is the most prominent consideration, as it can cause severe damage. The force of impact on the bridge abutment during a debris flow is significant and can lead to tilting, displacement, and even collapse of the bridge abutment. If the clearance area under the bridge is insufficient and the drainage capacity is small, serious siltation will occur under the bridge, which can pose a serious threat to safety if not cleaned up in time^41^. Therefore, when carrying out the design and construction of bridges, it is essential to reserve adequate overflow space under the bridge to mitigate and prevent damage. Corresponding prevention and control principles are thus set out for different risk areas.

The clearance area of a bridge is determined by its clear height and width. The clear height results from the combined consideration of the maximum accumulation thickness of the debris flow, the mud depth, the wash-up height, and the safety height. The formula is as follows:

14 $$H_{c} = d_{c} + H + \Delta H + h$$

where $H_{c}$ Indicates the net height of the bridge; $h$ indicates the safe height of the bridge (taken as 5.5 m); the rest of the values are the same as those described above.

The net width is related to the debris flow cross-section and the maximum particle size of the channel.

15 $$A = H_{C} \times B_{C}$$

16 $$D \ge (2\sim 4.5)D_{m}$$

where $A$ is the mudstone flow overflow area; $D_{m}$ is the maximum rock particle size (Fig. 2—the maximum rock particle size of the gully is 2.5 m, and the clear width of the proposed highway should not be less than 5.0 m).

The calculated minimum clear height and width values of the bridges are shown in Table 9.

**Table 9:** Calculation table of minimum clear height and minimum clear width required to ensure the safety of the proposed highway bridge.

Cross-section location	N 30° 2′ 29.23″ E 100° 58′ 26.71″ H: 2793 m	N 30° 03′ 00.78″ E 100° 57′ 30.07″ H: 2950 m	N 30° 02′ 53.41″ E 100° 57′ 57.45″ H: 2877 m	N 30° 03′ 06.95″ E 100° 57′ 03.28″ H: 3002 m
$H_{c} (m)$	14.58	14.59	16.36	16.3
$B_{c} (m)$	5.0	5.0	5.0	5.0

**Figure 13:**
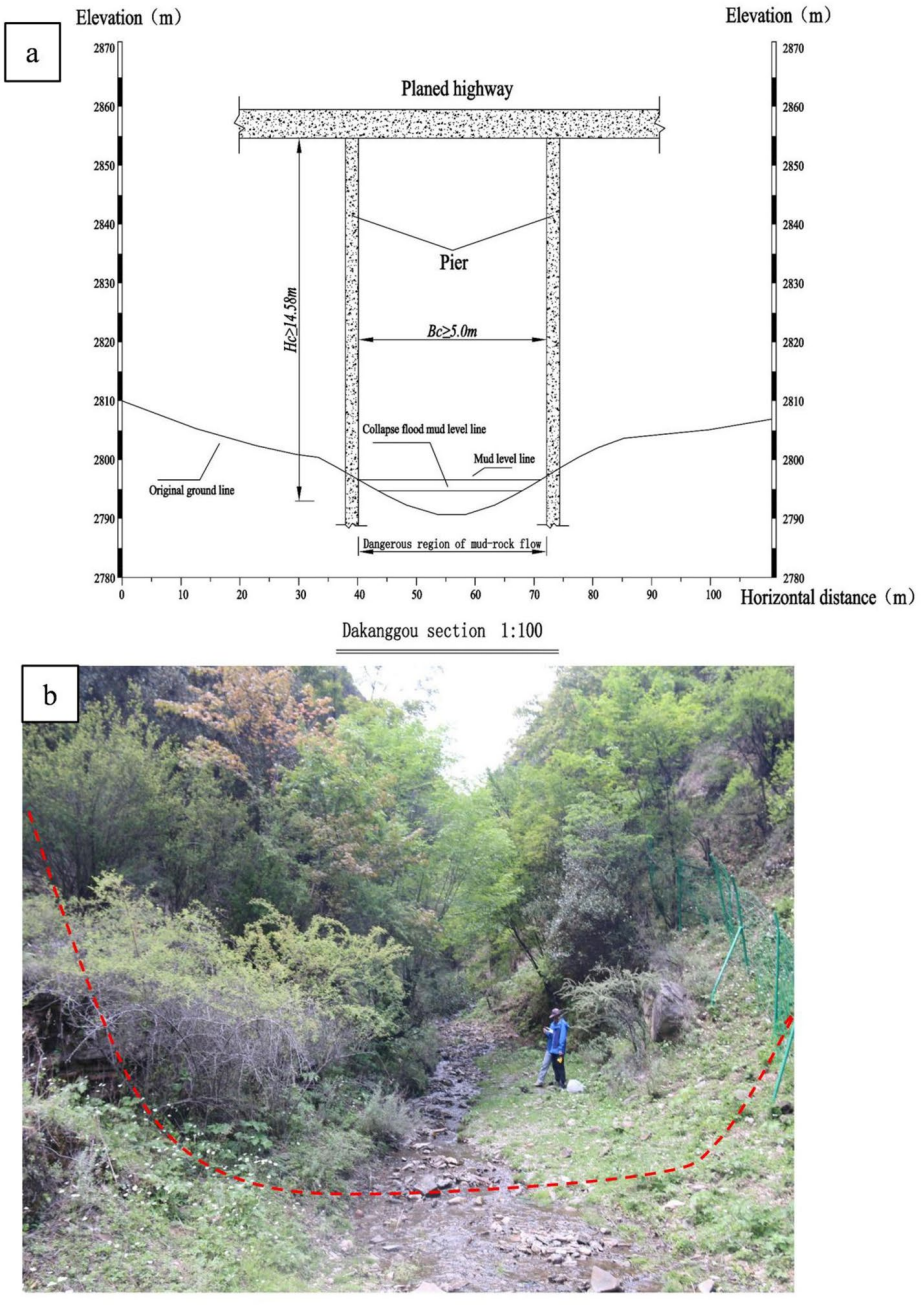
The site location and profile of the proposed bridge (Dakanggou section as the example).

The design results are shown in Fig. 13.

## Conclusions

1. This study proposes a method for the rapid evaluation of gully information entropy, the watershed unit material response rate, the degree of debris flow development, and road and debris flow danger based on geomorphic evolution theory coupled with material source sensitivity. The results show that the hazard risk level in the Dakang gully and 1# gully areas is medium, while the hazard risk level in the Ertuo gully and Yazhou gully areas is high.
2. The results of the debris flow hazard evaluation, which consider both the topography and physical sources, align well with the actual situation. They can objectively respond to the sizes of the debris flow hazards and provide more accurate results compared to using only the information entropy of the gully and the slope response rate of the physical sources. The evaluation of highway debris flow hazards, combining considerations of the degree of development of the debris flow and the location of the highway, is a more scientific and reasonable approach.
3. According to the morphological characteristics and engineering geological conditions of the debris flow in the Dakang, 1#, Yazhou, and Wutuo gullies, the location of the proposed bridge was selected. Based on satellite remote sensing images and field investigations, through the analysis and calculation of debris flow-related parameters, it was concluded that the maximum impact ranges of debris flows with a frequency of once in 100 years (P = 1) were 9.08 m, 9.09 m, 10.86 m, and 10.08 m for the Dakang, 1#, Yazhou, and Wutuo gullies, respectively. Based on a safe bridge height of 5.5 m, the recommended safe clearance heights for the Dakang, 1#, Yazhou, and Wutuo gullies should be 14.58 m, 14.59 m, 16.36 m, and 16.3 m, respectively. The safe clearance width for all four gullies should be 5.0 m.

## Data Availability

All data generated or analyzed during this study are included in this published article.
